# Workplace violence from the ambulance personnel’s perspective, a qualitative study

**DOI:** 10.1186/s13049-026-01680-8

**Published:** 2026-08-25

**Authors:** Magnus Viking, Karin Hugelius, Erik Höglund, Lisa Kurland

**Affiliations:** 1 Department of Ambulance Service, Faculty of Medicine and Health, Örebro University, Örebro, Sweden; 2 Faculty of Medicine and Health, Örebro University, Örebro, Sweden

**Keywords:** Ambulance care, Workplace violence, Ambulance personnel, Thematic analysis, Qualitative study

## Abstract

**Background:**

Despite increasing interest in the occurrence of workplace violence in ambulance services, few studies have explored the phenomenon from the perspective of the ambulance personnel. While previous studies have identified its incidence and risk factors, this study aimed to explore ambulance personnel’s understanding of why workplace violence occurs.

**Methods:**

An inductive, qualitative study was conducted using digital focus group interviews. The inclusion criteria were as follows – having worked in ambulance services for a minimum of two years and having personally experienced workplace violence. Reflexive thematic analysis was employed in this study.

**Results:**

Three focus group interviews with 10 Swedish ambulance personnel were included in the study. Three themes were identified: (I) ambulance personnel’s lack of competence and willingness to adapt can lead to workplace violence; (II) ambulance personnel’s inability to cope with their emotional response to provocation can lead to workplace violence; and (III) experiencing limited control in unpredictable situations. Workplace violence occurred when the ambulance personnel lacked empathy and compassion for the patient, acted disrespectfully, or when the power imbalance between ambulance personnel and the patient became apparent. If ambulance personnel felt provoked by the patient’s behaviour, it triggered workplace violence. Furthermore, if they could not read the situation and lost control of it or if the patient’s behaviour was unpredictable (e.g. if the patient was under the influence of drugs or alcohol), workplace violence ensued.

**Conclusions:**

Ambulance personnel need to acknowledge their own contributions to the risk of workplace violence and identify situations and actions that could both increase and decrease the risk of exposure. Future studies should focus on implementing interventions to identify potential high-risk situations and train ambulance personnel to deescalate risky situations.

**Supplementary Information:**

The online version contains supplementary material available at https://doi.org/10.1186/s13049-026-01680-8.

## Background

Ambulance personnel provide emergency care for patients with a wide plethora of conditions in addition to performing medical assessments in a multitude of environments at all times of the day and year. The working environment is often unpredictable, and ambulance missions hold an inherent risk of workplace violence [1, 3]. The risk of being exposed to workplace violence in ambulance services has been reported to occur in 0.4%–5% of ambulance missions [1, 2, 4, 7]. Workplace violence is commonly categorised as physical or non-physical violence [8, 9] and exposure to non-physical violence is the most common [1, 2, 10]. When exposed to violence, ambulance personnel can experience psychological distress, anxiety and fear [11]. These emotional reactions can also lead to negative consequences for patients, as the quality of care provided may be compromised [11, 12]. Hence, although workplace violence is relatively uncommon, its effects are disadvantageous for both patients and ambulance personnel.

The reason workplace violence occurs within ambulance services is known only to some extent. External factors related to the environment, including stress and situations when the patient perceives physical discomfort, have been shown to contribute to the development of violence [10, 13]. Additionally, patient characteristics, such as having an impulsive personality or suffering from mental health issues, have been shown to increase the risk of workplace violence [1, 14, 15]. Importantly, several studies highlight that ambulance personnels’ behaviour may trigger workplace violence, e.g. by disrespecting the patient or by having different expectations for the care being offered [11, 13, 16]. Although prior studies have mapped both incidence and risk factors, few studies have focused on why workplace violence arises from the perspective of ambulance personnel. Therefore, this study aimed to explore ambulance personnels’ understanding of why workplace violence occurs.

## Methods

### Design

The design was a qualitative focus group interview study with reflexive thematic analysis [17]. The RTARG checklist was used [18, 19].

A constructivist paradigm with a relativist ontology [20] was adopted, viewing reality as multiple and shaped by lived experience. Knowledge is in this context understood as socially constructed through participant–researcher interaction, making qualitative, semi-structured focus groups suitable for exploring ambulance personnel’s experiences of workplace violence.

### Reflexive considerations

Using reflexive thematic analysis requires the authors to acknowledge their researcher subjectivity and understanding of the phenomenon [17]. The authors have extensive clinical experience as ambulance nurses and emergency physicians. The research team have experience in emergency medicine, including prehospital research and have prior experience of studies relating to the phenomenon of workplace violence within the ambulance services [1, 11, 13]. Both clinical and scientific experience within the team informed the study design, in addition, the results from our prior study [11] indicated that something in the interaction between the ambulance personnel and the patient could lead to workplace violence. These experiences added value to the data collection procedures, including the first author´s (MV) previous experiences of conducting interviews on workplace violence within the ambulance service [11]. When designing the study, the authors’ preunderstanding, both clinical and scientific, helped form the research question and the interview guide. The interview guide was developed based on results from prior studies [1, 11, 13], but the research team was open to other explanations to why workplace violence might occur. The interview guide served to stay focused and aligned with the research question. Both clinical and research experience enabled the interviewer (MV), to build trust, and make the study participants comfortable. Also, this reduced the need for the study participants to clarify specific expressions used within the ambulance services, reducing the risk for unnecessary interruptions of the dialogues. When identifying patterns and creating themes, the authors were aware of how their experiences may have influenced the analysis and used continual reflection and discussion to ensure a valid interpretation of the data.

### Study setting

The Swedish ambulance service is part of the public healthcare system. Ambulances are typically staffed by a team of two professionals, where at least one is a registered nurse [21], often specialised in prehospital emergency care. The second crew member can be a registered nurse or an emergency medical technician.

### Study participants

The study participants were identified through purposeful sampling [22], as they were required to have the following criteria (1) at least two years’ experience of working in the ambulance service and (2) personal experience of workplace violence. An invitation to participate was sent by e-mail to the head of the ambulance services in seven of the 21 regions in Sweden with the intention of recruiting participants from both rural and urban areas. The head of the ambulance service in each region was asked to verbally inform and distribute the study information, using a study flyer provided by the research team. The study flyer included study and contact information which the ambulance personnel used if they were interested in participating. The research team included ambulance personal who were positive to participate and met the inclusion criteria. Interview groups were formed with the intention to include study participants from different regions, and when possible, include both men and women in each group.

The inclusion criteria were ambulance personnel with at least two years of experience working in the ambulance service and with prior personal exposure to workplace violence. The exclusion criterion was ambulance personnel who lacked the technical ability to participate in digital interviews. Study participants who voluntarily expressed interest to participate, and who could participate in the times offered, were included, regardless of sex, age or years of experience. All study participants gave their informed consent verbally before participating in the interviews.

### Data collection

A semi-structured interview guide developed by the research team and based on previous studies was used (see Supplementary file 1) [1, 11]. A pilot focus group discussion with three participants was conducted to test the interview guide, which was not changed after the pilot interview. The interview guide was used as a semi-structured, flexible supporting tool to ensure that all topics were addressed in all interviews. However, the questions were not necessarily asked in a fixed sequence, allowing the interviewer to follow the flow of the conversation and to ask follow-up questions that emerged from the discussions. The pilot interview was not included in the current study.

Three digital focus group interviews were conducted with three to four participants between 23 October and 26 November 2024. Participants were allocated to focus groups based on their availability. The interviews were led by the first author (MV) using a digital video streaming platform (Visiba Care - web and app version 2024). The interviews were audio recorded using OBS Studio (30.2.3) and transcribed by a professional transcribing service.

### Analysis

A latent reflexive thematic analysis [18, 19] of the data was conducted. The analysis was performed in six steps. (1) All authors read the transcribed interviews several times. This allowed the authors to familiarise themselves with the data. (2) Thereafter, the data was coded by the first author (MV). After reading and reflecting on the content several times, recoding was done to understand the underlying meaning of the participants’ discourse. As the coding began, the positionality of the authors, with long clinical experiences within the ambulance services, was used to recognize and understand patterns in the narratives analysed. The clinical experience contributed to a deeper understanding of certain expressions and underlying meanings of the narratives in relation to the research question. All the authors contributed to this step. (3) The third step was to create themes from the codes. Themes were discussed and reflected on by all authors and revised several times to ensure that they were firmly grounded in the data. When creating and analysing the themes, thematic maps were used to acquire an overview of the data, the codes and their relationship. (4) Thereafter, the authors reviewed and created the themes. This step was an iterative process involving close collaboration among all the authors. Additionally, the first author reread the interview transcripts to ensure that the themes accurately reflected the data. (5) During this step, the final themes were defined and named. (6) The final step of the analysis was to write the manuscript.

### Ethical considerations

The study was approved by the Swedish Ethical Review Authority (2023-06503-02 and 2024-06031-02). Participants received both written and verbal study information. Informed consent was collected verbally prior to the interviews. Considering the defined population of ambulance personnel, there could be a potential risk of participant identification. To minimise this, identifying details were limited and quotations were selected and reviewed with careful consideration to avoid indirect identification.

## Results

This study comprised 10 participants. Two interviews included three participants, and one interview included four participants. The participants gathered from five different regions in Sweden and reflected on their experiences working as ambulance personnel in both large cities and urban areas. One of the invited regions declined to participate and from one of the invited regions, no participant volunteered. The length of the interviews ranged from 1 h to 22 min to 1 h and 33 min. The demographics of the participants are shown in Table 1.

**Table 1:** Participant demographics

Demographic		
Sex, n	Women/Men	8/2
Age, in years, mean (min – max)		44 (31–60)
Profession, n	RN, specialised in prehospital emergency care	6
	RN	3
	EMT	1
Years of experience within ambulance services, mean (min – max)		15 (2–36)

RN - Registered Nurse, EMT - Emergency Medical Technician

### Themes

Three themes described the ambulance personnel’s understanding of why workplace violence occurred. The content of each theme reflects triggers for workplace violence, either on their own or in combination with components from other themes.

### Ambulance personnel’s lack of competence and willingness to adapt can lead to workplace violence

This theme reflects ambulance personnel’s unwillingness to adapt to patients’ needs, and to a situation that could lead to aggressive behaviour from patients.

Lacking the willingness to meet patients with empathy and compassion may sometimes hinder the formation of a relationship and a professional interaction between the patient and the ambulance personnel, making the patient feel disrespected and triggering aggressive behaviour. In addition, disrespecting patients’ religious or cultural beliefs or customs could trigger workplace violence.

- You need to be a social chameleon, and read what kind of people who are in the room, what kind of socio-economic group they are from, where they originate from, what kind of cultural background they have, and what kind of body language they use […] For me, I had grown up with this kind of older men, and I understood that it was the personal sphere, that was the problem (Interview 1, Interviewee 1).

Ambulance personnel who are willing to adapt to the patient and the specific situation could, at times, de-escalate an aggressive situation. If the ambulance personnel lose their professional composure and acts without empathy and compassion, it can provoke the patient and lead to workplace violence.

- And my colleague in the driver’s seat, she liked to provoke the patient in many ways, but the patient chose to play unconscious. Eventually, the patient couldn’t take it anymore because my colleague was shouting things. Suddenly, they started shouting at each other. The patient was lying on the stretcher, and I was sitting behind her. My colleague was driving the car, and […] she thought it was very funny at the time, but the patient […] she was really provoked by this. (Interview 3, Interviewee 2)

An understanding of the power imbalance between ambulance personnel as caregivers and the patient or family member is essential to either trigger or reduce the occurrence of workplace violence. Ambulance personnel perceived that their inherent superiority in relation to the patient could be perceived as provocative by the patient. Power imbalances could lead to an aggressive response in situations where the patient was not allowed to influence decisions about their medical treatment or care options or if ambulance personnel were required by law to notify authorities, e.g. the social services.

- […] you need to have an understanding of and respect for the power we [the ambulance personnel] have in the encounter, because that’s what triggers the situation and that you are aware that it’s me who will make the decisions in this situation […] that’s the fine-tuning in these situations, to not use power because it will trigger the situation […] that you take some time and don’t force things, because that will make the patient snap. (Interview 3, Interviewee 3)

If the ambulance personnel did not confirm the individual, i.e. recognize and respond to the individual’s needs, or achieve a good relationship with them, the patient could become aggressive. Also, if the ambulance personnel’s body language signalled superiority or if they did not recognise the patient as an individual, it could provoke the patient and result in aggression. At the same time, because a lack of compassion and empathy could trigger violent behaviour, the ambulance personnel sometimes could be perceived as overly trusting, failing to appreciate the patient’s perspective or the human dynamics involved in the situation. This could render them naïve, approaching each scene and patient with the intention of ‘doing good’ and not being prepared for meeting aggressive patients. If ambulance personnel gain information about previous aggressive behaviour, they could prepare themselves and use strategies and behaviours to mitigate such risks.

- […] I’ve never seen so many warnings in the medical records, meaning that he had been on trial because he was violent, really violent, towards healthcare personnel. I don’t know if it would have been good or bad [to read the information before the patient interaction]. […] Maybe we would have handled the situation a little differently if we had opened that medical record beforehand. (Interview 3, Interviewee 2)

The will to ‘do good’, to form a relationship and to care for the patient on equal terms, could often be protective. It reduced the risk of triggering violent behaviour from the patient but could, at times, also increase the risk of workplace violence, since the ambulance personnel tend to be willing to put safety measures aside. Thus, they became more vulnerable.

### Ambulance personnel’s inability to cope with their own emotional response to provocation can lead to workplace violence

This theme describes insights reflecting the emotions that arise when ambulance personnel are provoked by something in their interactions with the patient. Examples of such emotions are frustration, disappointment and feelings of being disrespected.

When ambulance personnel are provoked by a patient’s or relative’s behaviour or disappointed when the situation does not require their competence or services, they can fail to balance their emotions and act unprofessionally. This response includes directing their frustration towards the patient or a family member or expressing their frustration verbally. Such an unprofessional reaction and behaviour by ambulance personnel could trigger aggressive behaviour from the patient or their family members.

- I was really triggered by this man, who became very threatening because he didn’t want to comply. And I know that’s a really big trigger for me, and I am aware of it, and I try to back off and take a deep breath […] and then I just react and then it becomes a spiral of emotions and feelings. (Interview 3, Interviewee 4)

Ambulance personnel could perceive both personal and societal expectations when serving the community and people in need of care, as well as in situations where workplace violence could occur and described discomfort when being unable to provide what they perceived as necessary care. A strong will to be of help can therefore cause ambulance personnel to engage in situations at a higher risk of workplace violence. Their moral and underlying expectations to save lives could make the ambulance personnel accept an increased risk of workplace violence.

- There is also an incredible amount of ethics involved in this. I don’t know if I would have been able to sleep if he jumped out the window, he was out with one leg and […] I managed to pull him back with brute force. But man, you must be able to live with the fact that he had jumped […] And that […] it is a very high degree of putting ourselves in harm’s way and choosing or feeling forced or urged, ethically, to care for a patient who has threatened to kill you. (Interview 3, Interviewee 3)

### Experiencing limited control in unpredictable situations

This theme describes ambulance personnel’s experiences of limited control in unpredictable situations. Seemingly calm encounters could escalate into aggression when warning signs were unclear or difficult for the ambulance personnel to recognise.

In some cases, ambulance personnel found it difficult to interpret patients’ intentions, particularly when patients were affected by substances or severe mental illness. These conditions could hinder communication and the establishment of mutual understanding, which in turn could lead to frustration and, at times, escalation into violent behaviour.

- … the patient is so mentally ill that she […] well, she is delusional, you can’t have a conversation with her, so she is really, really sick […] there was no way of communicating because the patient wasn’t receptive to speech. She was in her own little world. But she experienced me as threatening […], so when I started talking to her and asked how she was feeling, she hit me—out of nowhere. (Interview 2, Interviewee 1)

Especially when ambulance personnel lack experience of how to deal with hostile patients, the risk of losing control could be high.

- It is also about experience, you make demands and say “no” more often. I think it’s more often the case that when you’re new in the ambulance service, you are more exposed to workplace violence. (Interview 1, Interviewee 1)
- I think so too, because I believe this profession is a bit of ‘learning by pain’ and it is hard to teach someone how to act and think. Unfortunately, the best lesson is … that not everyone is an angel that you can come in and save. (Interview 1, Interviewee 2)

Ambulance personnel described that they could arrive on scene to a patient that was already stressed or agitated. The patient could be agitated because of events or things that had happened prior to the ambulance personnel’s arrival and could at times expose the ambulance personnel to unprovoked and unexpected violence.

- There was a dispatch that the patient was about to take her life, and the fire department was first on scene and had broken down the door. When we arrived and I [the ambulance personnel] was going to be nice and say, ‘Hello, how are you doing?’, I received a blow. [It came] out of nowhere […] everything happened so fast – she [the patient] didn’t understand why people were breaking down [her] door. (Interview 2, Interviewee 1)

At times, ambulance personnel described a window of opportunity to prevent escalation by adapting their response. This required clinical experience and skills to interpret patient cues and respond appropriately.

## Discussion

The ambulance personnel’s understanding of why workplace violence occurs was described in three themes, as t*he ambulance personnel’s lack of competence and willingness to adapt*,* the ambulance personnel’s inability to cope with their emotional response to provocation* and *experiencing limited control in unpredictable situations can lead to workplace violence.* The circumstances reflected in these themes can, both individually and in combination, lead to workplace violence.

This study showed that when ambulance personnel were unable to adapt to patients’ needs and to the situation, this could trigger aggression from the patient. Similar findings have been reported previously, both within ambulance services [23] and in other healthcare contexts [24]. Prior research describes that ambulance personnel tend to act disrespectfully towards patients due to working conditions and stressors in the work environment, such as working in public places and communication problems with other health care organisations, which can result in a lack of compassion and empathy towards the patient [25]. Being professional constitutes clinical knowledge and skills, as well as awareness of attitudes and ethics in the patient interaction [26]. Additionally, ambulance personnel need competence, defined as the ability to apply knowledge and skills in specific contextual situations [27]. To support ambulance personnel in maintaining professional and compassionate behaviour, there is a need for organisational, collegial and managerial support and to retain ambulance personnel’s competence, compassion and empathy [28]. As described in the results, ambulance personnel experience both emotional and ethical challenges in relation to patient interactions. By facilitating a healthy work environment and well-functioning workplace structures, these challenges could be alleviated [29], potentially contributing to a reduction in workplace violence.

The results also indicate that ambulance personnel were at times unable to cope with their emotional response to provocation caused by the misalignment between them and the patient’s expectations. This disaccord between ambulance personnel and patients has been described previously [30, 31]. The ambulance personnel expect to meet patients with emergency medical needs [32]. If ambulance personnel feel their services are being misused [32], they may become disrespectful towards the patient, potentially triggering workplace violence [13]. Since an increasing number of patients seek ambulance care for non-acute medical conditions [33], such perceived provocations may increase. Therefore, it is important that ambulance personnel be trained to handle their own emotions so as not to be triggered by such situations.

The results suggest that limited control in unpredictable situations is a condition under which the risk of workplace violence may arise. The risk is accentuated since ambulance services operate in complex and often unpredictable contexts. The results of this study outline such an unpredictable context when ambulance personnel at times perceive difficulties interacting with patients who suffer from mental health issues. Recent studies have found that these patients have an increased risk of exposing ambulance personnel to workplace violence [1, 2]. However, it is also important to keep in mind that only a small proportion of patients with mental health issues act aggressively [1]. The ambulance personnel need to be competent in reading the situation, which would enable them to make quick and correct decisions and, when required, change their behaviour, thereby adapting to the situation [34, 35]. Being able to read the situation stems from having situational awareness, which can be obtained at different levels, with the lowest levels focusing on what is happening presently [36]. Ambulance personnel can develop a higher level of situational awareness through experience and training, which will enable them to identify signs of risk for workplace violence and predict future developments [36]. A high level of situational awareness is necessary to be able to take the necessary actions to minimise the risk of losing control. Ambulance personnel have been shown to improve their ability to develop situational awareness by approximately 20% within as little as one hour of training [36, 37]. Therefore, professional competence, including the understanding of and capacity to apply situational awareness in unexpected situations, could enable safer working conditions. Future research should focus on exploring how the concept of situational awareness is used in situations where workplace violence occurs.

### Limitations

This study builds on data collected through three focus group interviews with 10 participants. It is possible that with the use of focus groups, some participants did not get the opportunity to speak their minds, share certain experiences with other study participants or even participate in the interviews. However, the use of focus group interviews to collect data was chosen for its ability to make the participants generate their own questions when discussing with each other, providing a richer and more nuanced description of the phenomenon and their experiences, which revealed a deeper dimension of information [38].

The size of the focus groups may be considered a limitation. However, the small number of study participants in each focus group facilitated in-depth discussions and meaningful interaction. The authors observed that the participants appeared comfortable sharing even unpleasant experiences within the smaller group setting [39, 40].

When using reflexive thematic analysis, there is no fixed number of study participants required. Instead, the concept of information power [41] was applied in this study. However, it cannot be ruled out that a larger or different study sample would have yielded different results. The number of participants was guided by the narrow aim and study sample, where all study participants had professional experience of being exposed to workplace violence, including criteria that implied that all study participants had personal experiences with the phenomena studied. Furthermore, information power was achieved through high-quality focus group dialogues led by a dialogue leader with professional experience in ambulance services and in conducting research interviews. All authors had clinical experience in ambulance services and emergency care, as well as research experience on workplace violence in ambulance services, which added value to the analysis and information power [42].

Credibility was enhanced through the author’s understanding of the field, through multiple researchers working together in the analysis of the data and through double-checking the findings against the raw data [43]. The data were collected in a Swedish context and the study sample included ambulance personnel working in both larger cities and rural areas. However, given the substantial differences in how ambulance services are organised [44] and the role of the ambulance services from a global perspective, it may be discussed how valid the results are in other cultural and organisational contexts. Despite these differences, the meeting between the patient and ambulance personnel relies on human interaction, and therefore, this study may still add value to understanding this interaction in relation to workplace violence. As in all qualitative studies, it cannot be excluded that with other study participants and another research team, that the results would have been somewhat different.

## Conclusion

Ambulance personnel’s understanding of why workplace violence occurs can be described under three themes as t*he ambulance personnel’s lack of competence and willingness to adapt*,* the ambulance personnel’s inability to cope with their emotional response to provocation* and *experiencing limited control in unpredictable situations*. Ambulance personnel need to acknowledge their own contribution to the risk of workplace violence and which situations and actions could increase this risk. Further study is needed to understand the effect of interventions, e.g., reflection, peer support and organisational efforts, including training on how and when to react to situations that might become aggressive.

## Supplementary Information

Below is the link to the electronic supplementary material.


Supplementary Material 1


## Data Availability

The data that support the findings of this study are available from Region Örebro county, but restrictions apply to the availability of these data, which were used under licence for the current study and so are not publicly available. The data are, however, available upon request and with the permission of the Swedish Ethical Review Authority.
