# Supplementary material for: Workplace violence from the ambulance personnel’s perspective, a qualitative study
Source: Scand J Trauma Resusc Emerg Med. 2026 Aug 25;34:146. doi: 10.1186/s13049-026-01680-8 (PMC13531993; doi:10.1186/s13049-026-01680-8)
Supplement: Supplementary file 1 [file 13049_2026_1680_MOESM1_ESM.docx]

1. Supplementary file – Semi-structured questionnaire

The WASP-project; Workplace violence in the ambulance service

Region Örebro county/ Örebro university

2023

**Interview guide**

Based on previous studies within this project we have gained knowledge of the incidence of workplace violence against ambulance personnel and how they perceive being exposed to workplace violence. For example, have we been able to see that the perpetrator most often is a patient, man and at many of the situations they were under the influence of alcohol or drugs, or suffered from mental illness.

Previous results also show that the ambulance personnel experience situations where they been exposed to workplace violence as very unpleasant and that they used different approaches to handle the situations. It varies from attempts of de-escalation of aggressiveness through certain behaviour to a fight or flight behaviour.

Even though we have gained much insight into workplace violence, we also acknowledge that we have limited knowledge about what happens in the patient interaction between the patient and the ambulance personnel. It seems that things happen in that interaction that wasn’t meant to happen but still happened. That is something we want to know more about and that is why I want to talk to all of you that has experience of situations with workplace violence in the ambulance service.

1. All of you have at some or several points in your work as ambulance personnel been exposed to workplace violence. If you think about one situation you been in where workplace violence occurred.

- What happened in that patient interaction?
- Why did workplace violence occur?
- Which difficulties did you face in the patient interaction?
- What was most important for you in the patient interaction?

2. If we were to discuss more generally about situations containing workplace violence.

- Is there something in the patient interaction that you would say increase the risk for workplace violence to occur?
- What is your take on power disparities in the patient interaction with patients that are violent or aggressive?
- Where would you as ambulance personnel draw the line for what you would tolerate?
- Do you think your colleagues think in the same way?
- Has there been a change of time in this matter?

3. From previous research we know that when you have been exposed to workplace violence, you tend to be more vigilant to signs of workplace violence.

- Why do you think it is like that?
- Have you any experience of situations where you have been able to de-escalate or turn a situation with potential of workplace violence?
- Which advice would you give to new ambulance personnel about the interaction with patients or next of kin that act unpleasantly or aggressive, or where workplace violence occurred?

Follow up questions for more comprehensive answers:

- Can you tell me more?
- How do you reason in that regard?
- Can you elaborate?
- Can you elaborate me more about the context?
